# Supplementary material for: HIVprotI: an integrated web based platform for prediction and design of HIV proteins inhibitors
Source: J Cheminform. 2018 Mar 9;10:12. doi: 10.1186/s13321-018-0266-y (PMC5845081; doi:10.1186/s13321-018-0266-y)
Supplement: Supplementary file 2 — Additional file 2. Source code of HIVProtI web server. [file 13321_2018_266_MOESM2_ESM.zip › HIVprotI_Source-code/hivprotI/weka-3-6-6/doc/weka/core/neighboursearch/kdtrees/KDTreeNodeSplitter.html]

KDTreeNodeSplitter


---


|  |  |  |
| --- | --- | --- |
| **Overview** \| **Package** \| **Class** \| **Tree** \| **Deprecated** \| **Index** \| **Help** \| **Weka's home** | |  |
| **PREV CLASS**   **NEXT CLASS** | **FRAMES**    **NO FRAMES**     **All Classes** |  |
| SUMMARY: NESTED \| FIELD \| CONSTR \| METHOD | DETAIL: FIELD \| CONSTR \| METHOD |  |


---


## weka.core.neighboursearch.kdtrees Class KDTreeNodeSplitter

```
java.lang.Object
  weka.core.neighboursearch.kdtrees.KDTreeNodeSplitter
```

**All Implemented Interfaces:**: java.io.Serializable, OptionHandler, RevisionHandler

**Direct Known Subclasses:**: KMeansInpiredMethod, MedianOfWidestDimension, MidPointOfWidestDimension, SlidingMidPointOfWidestSide

---

``` public abstract class KDTreeNodeSplitter extends java.lang.Object implements java.io.Serializable, OptionHandler, RevisionHandler ```

Class that splits up a KDTreeNode.

**Version:**
:   $Revision: 1.2 $

**Author:**
:   Ashraf M. Kibriya (amk14[at-the-rate]cs[dot]waikato[dot]ac[dot]nz)

**See Also:**: Serialized Form

---

| **Field Summary** | |
| --- | --- |
| `static int` | `MAX`             Index of max value in an array of attributes' range. |
| `static int` | `MIN`             Index of min value in an array of attributes' range. |
| `static int` | `WIDTH`             Index of width value (max-min) in an array of attributes' range. |


**Constructor Summary**

`KDTreeNodeSplitter()`
  
          default constructor.

`KDTreeNodeSplitter(int[] instList,
Instances insts,
EuclideanDistance e)`
  
          Creates a new instance of KDTreeNodeSplitter.


| **Method Summary** | |
| --- | --- |
| `java.lang.String[]` | `getOptions()`             Gets the current settings of the object. |
| `java.lang.String` | `getRevision()`             Returns the revision string. |
| `java.util.Enumeration` | `listOptions()`             Returns an enumeration describing the available options. |
| `void` | `setEuclideanDistanceFunction(EuclideanDistance func)`             Sets the EuclideanDistance object to use for splitting nodes. |
| `void` | `setInstanceList(int[] instList)`             Sets the master index array containing indices of the training instances. |
| `void` | `setInstances(Instances inst)`             Sets the training instances on which the tree is (or is to be) built. |
| `void` | `setNodeWidthNormalization(boolean normalize)`             Sets whether if a nodes region is normalized or not. |
| `void` | `setOptions(java.lang.String[] options)`             Parses a given list of options. |
| `abstract  void` | `splitNode(KDTreeNode node, int numNodesCreated, double[][] nodeRanges, double[][] universe)`             Splits a node into two. |

**Methods inherited from class java.lang.Object**

`equals, getClass, hashCode, notify, notifyAll, toString, wait, wait, wait`

**Field Detail**

### MIN

```
public static final int MIN
```

:   Index of min value in an array of attributes' range.

    **See Also:**: Constant Field Values

---


### MAX

```
public static final int MAX
```

:   Index of max value in an array of attributes' range.

    **See Also:**: Constant Field Values

---


### WIDTH

```
public static final int WIDTH
```

:   Index of width value (max-min) in an array of attributes' range.

    **See Also:**: Constant Field Values


**Constructor Detail**

### KDTreeNodeSplitter

```
public KDTreeNodeSplitter()
```

:   default constructor.

---


### KDTreeNodeSplitter

```
public KDTreeNodeSplitter(int[] instList,
                          Instances insts,
                          EuclideanDistance e)
```

:   Creates a new instance of KDTreeNodeSplitter.

    **Parameters:**: `instList` - Reference of the master index array.: `insts` - The set of training instances on which the tree is built.: `e` - The EuclideanDistance object that is used in tree contruction.


**Method Detail**

### listOptions

```
public java.util.Enumeration listOptions()
```

:   Returns an enumeration describing the available options.

    :   **Specified by:**: `listOptions` in interface `OptionHandler`
    :   **Returns:**: an enumeration of all the available options.

---


### setOptions

```
public void setOptions(java.lang.String[] options)
                throws java.lang.Exception
```

:   Parses a given list of options.

    :   **Specified by:**: `setOptions` in interface `OptionHandler`
    :   **Parameters:**: `options` - the list of options as an array of strings **Throws:**: `java.lang.Exception` - if an option is not supported

---


### getOptions

```
public java.lang.String[] getOptions()
```

:   Gets the current settings of the object.

    :   **Specified by:**: `getOptions` in interface `OptionHandler`
    :   **Returns:**: an array of strings suitable for passing to setOptions

---


### splitNode

```
public abstract void splitNode(KDTreeNode node,
                               int numNodesCreated,
                               double[][] nodeRanges,
                               double[][] universe)
                        throws java.lang.Exception
```

:   Splits a node into two. After splitting two new nodes are created
    and correctly initialised. And, node.left and node.right are
    set appropriately.

    :   **Parameters:**: `node` - The node to split.: `numNodesCreated` - The number of nodes that so far have been created for the tree, so that the newly created nodes are assigned correct/meaningful node numbers/ids.: `nodeRanges` - The attributes' range for the points inside the node that is to be split.: `universe` - The attributes' range for the whole point-space. **Throws:**: `java.lang.Exception` - If there is some problem in splitting the given node.

---


### setInstances

```
public void setInstances(Instances inst)
```

:   Sets the training instances on which the tree is (or is
    to be) built.

    :   **Parameters:**: `inst` - The training instances.

---


### setInstanceList

```
public void setInstanceList(int[] instList)
```

:   Sets the master index array containing indices of the
    training instances. This array will be rearranged as
    the tree is built, so that each node is assigned a
    portion in this array which contain the instances
    insides the node's region.

    :   **Parameters:**: `instList` - The master index array.

---


### setEuclideanDistanceFunction

```
public void setEuclideanDistanceFunction(EuclideanDistance func)
```

:   Sets the EuclideanDistance object to use for
    splitting nodes.

    :   **Parameters:**: `func` - The EuclideanDistance object.

---


### setNodeWidthNormalization

```
public void setNodeWidthNormalization(boolean normalize)
```

:   Sets whether if a nodes region is normalized
    or not. If set to true then, when selecting
    the widest attribute/dimension for splitting,
    the width of each attribute/dimension,
    of the points inside the node's region, is
    divided by the width of that
    attribute/dimension for the whole point-space.
    Thus, each attribute/dimension of that node
    is normalized.

    :   **Parameters:**: `normalize` - Should be true if normalization is required.

---


### getRevision

```
public java.lang.String getRevision()
```

:   Returns the revision string.

    :   **Specified by:**: `getRevision` in interface `RevisionHandler`
    :   **Returns:**: the revision


---


|  |  |  |
| --- | --- | --- |
| **Overview** \| **Package** \| **Class** \| **Tree** \| **Deprecated** \| **Index** \| **Help** \| **Weka's home** | |  |
| **PREV CLASS**   **NEXT CLASS** | **FRAMES**    **NO FRAMES**     **All Classes** |  |
| SUMMARY: NESTED \| FIELD \| CONSTR \| METHOD | DETAIL: FIELD \| CONSTR \| METHOD |  |


---
